# Primary cultivation: factors affecting contamination and *Mycobacterium ulcerans* growth after long turnover time of clinical specimens

**DOI:** 10.1186/s12879-014-0636-7

**Published:** 2014-11-30

**Authors:** Martin W Bratschi, Miriam Bolz, Leticia Grize, Sarah Kerber, Jacques C Minyem, Alphonse Um Boock, Dorothy Yeboah-Manu, Marie-Thérèse Ruf, Gerd Pluschke

**Affiliations:** Swiss Tropical and Public Health Institute, Basel, Switzerland; University of Basel, Basel, Switzerland; FAIRMED Africa Regional Office, Yaoundé, Cameroon; Noguchi Memorial Institute for Medical Research, University of Ghana, Legon Accra, Ghana

**Keywords:** Buruli ulcer, Mycobacterium ulcerans, Primary cultivation, Long turnover time

## Abstract

**Background:**

While cultivation of pathogens represents a foundational diagnostic approach in the study of infectious diseases, its value for the confirmation of clinical diagnosis of Buruli ulcer is limited by the fact that colonies of *Mycobacterium ulcerans* appear only after about eight weeks of incubation at 30°C. However, for molecular epidemiological and drug sensitivity studies, primary isolation of *M. ulcerans* remains an essential tool. Since for most of the remote Buruli ulcer endemic regions of Africa cultivation laboratories are not easily accessible, samples from lesions often have to be stored for extended periods of time prior to processing. The objective of the current study therefore was to determine which transport medium, decontamination method or other factors decrease the contamination rate and increase the chance of primary isolation of *M. ulcerans* bacilli after long turnover time.

**Methods:**

Swab and fine needle aspirate (FNA) samples for the primary cultivation were collected from clinically confirmed Buruli ulcer patients in the Mapé Basin of Cameroon. The samples were either stored in the semi-solid transport media 7H9 or Amies or dry for extended period of time prior to processing. In the laboratory, four decontamination methods and two inoculation media were evaluated and statistical methods applied to identify factors that decrease culture contamination and factors that increase the probability of *M. ulcerans* recovery.

**Results:**

The analysis showed: i) that the use of moist transport media significantly increased the recovery rate of *M. ulcerans* compared to samples kept dry*;* ii) that the choice of the decontamination method had no significant effect on the chance of *M. ulcerans* isolation; and iii) that Löwenstein-Jensen supplemented with antibiotics as inoculation medium yielded the best results. We further found that, ten extra days between sampling and inoculation lead to a relative decrease in the isolation rate of *M. ulcerans* by nearly 20%. Finally, collection and processing of multiple samples per patient was found to significantly increase the *M. ulcerans* isolation rate.

**Conclusions:**

Based on our analysis we suggest a procedure suitable for the primary isolation of *M. ulcerans* strains from patients following long delay between sample collection and processing to establish a *M. ulcerans* strain collection for research purposes.

**Electronic supplementary material:**

The online version of this article (doi:10.1186/s12879-014-0636-7) contains supplementary material, which is available to authorized users.

## Background

Buruli ulcer (BU), a neglected tropical disease of the skin caused by *Mycobacterium ulcerans*, has been reported from over 30 countries worldwide with most cases occurring in West Africa. Clinically BU presents with both non-ulcerative lesions, such as nodules, plaques and edema, as well as ulcers. The major burden of the disease falls on children between 5 and 15 years of age [1],[2]. Despite intensive research efforts, both the reservoir and the mode of transmission of *M. ulcerans* have remained unclear [1]. Currently available methods for laboratory diagnosis of BU are microscopy, histopathology, PCR for the *M. ulcerans* specific insertion sequence 2404 (IS2404) or primary culturing. Based on the high sensitivity and specificity, the IS2404 PCR test is considered the gold standard in BU diagnosis [2]. Historically, BU was treated by wide excision of lesions and tissue samples could easily be obtained for laboratory diagnosis. Since the introduction of rifampicin and streptomycin combination therapy in 2004 [3], samples for laboratory testing are fine needle aspirates (FNA) taken from closed lesions and swab specimens taken from the undermined edges of ulcers [4].

Although primary culturing of *M. ulcerans* can provide a definitive BU diagnosis, the value of this method for primary diagnosis is strongly hampered by the fact that colonies take two to three months to appear and even under optimal conditions the sensitivity of the method is limited [5],[6]. For clinical diagnosis, culturing therefore only represents an auxiliary to other methods for the laboratory confirmation of BU. However, for studies on treatment efficacy, molecular epidemiology, and drug sensitivity, primary isolation of *M. ulcerans* remains crucial [7],[8].

To prevent overgrowth with other faster growing microorganisms, primary culturing of *M. ulcerans* requires decontamination of clinical samples prior to culture inoculation [6]. The commonly used decontamination methods use NaOH and HCl (Petroff and reverse Petroff method) or oxalic acid (OA). Although necessary to prevent overgrowth, all of these methods have been shown to have a detrimental effect on the viability of *M. ulcerans* [6],[9],[10]. As for the culturing media on which *M. ulcerans* is isolated, PANTA, a mixture of the antibiotics polymyxin B, amphotericin B, nalidixic acid, trimethoprim and azlocillin, as an additive to Löwenstein–Jensen media can be used to prevent the growth of a range of microorganisms but does not have an inhibitory effect on the growth of *M. ulcerans* [10].

Since for cultivation of *M. ulcerans* a sophisticated laboratory infrastructure is required, clinical specimens from BU patients often cannot be processed in a timely manner. It has been shown that from tissue specimens transported in semisolid transport medium, positive cultures can be obtained even if samples were kept at ambient temperature for more than two months [11]. For FNA samples transported in liquid transport medium containing PANTA and processed within 2 weeks, Eddyani *et al*. further found a sensitivity of *in vitro* culture of 17.6%, which was not significantly lower than the culture positivity rate of 25.0% obtained by culturing tissue specimens from the same patients [12]. Yeboah-Manu *et al*. observed culture sensitivities of 41.7% for FNA samples from non-ulcerative lesions and of 43.8% for swab samples if they were transported on ice in transport media containing PANTA and processed within 24 hours [13]. The same study also yielded similar results when comparing the Petroff and the OA decontamination methods [13]. Further in a subset of samples in the same study, 33.3% of swabs transported dry and processed between seven days and one month after collection were culture positive [13].

For the current study we set out to determine the best procedure for the cultivation of *M. ulcerans* from swab and FNA samples stored over extended periods of time prior to processing. Specifically, the objectives of the current study were to determine how transport medium, decontamination method, inoculated media, transport time or other factors decrease the contamination rate and increase the chance of primary isolation of *M. ulcerans*. The procedure devised here is suitable for the primary isolation of *M. ulcerans* strains from patients following long delay between sample collection and processing to establish a strain collection for research purposes.

## Methods

### Patient recruitment and ethical statement

For the current study samples were collected from BU patients attending health facilities in the Mapé Basin of Cameroon [14] between August 2010 and July 2012. Locally patients were diagnosed and treatment initiated based on clinical symptoms and if available Ziehl-Neelsen staining. Samples for laboratory confirmation were collected and processed as described below. Ethical clearance for the study was obtained from the Cameroon National Ethics Committee (N°041/CNE/DNM/09 and N°172/CNE/SE/2011) and the Ethics Committee of Basel (EKBB, reference no. 53/11). Participation was voluntary and all patients who participated in the study or their legal guardian provided written informed consent.

### Sample collection, storage and transport

Prior to the start of medical treatment FNA were collected from patients with non-ulcerative lesions and swabs from patients with ulcers. The number of swabs depended on the number of lesions, lesion size and clinical judgment. FNA were collected with sterile needles and swabs using individually packed sterile cotton swabs. To minimize the handling of needles and avoid any dilution of the samples in transport medium, FNA were transferred onto a cotton swab immediately after collection. These swabs were then processed the same as the swabs collected from ulcers and in the remainder of the manuscript we refer to all samples as swabs. Swabs were stored dry, in 7H9 medium containing PANTA (7H9) or in the antibiotics free Amies medium (VWR International); the latter two being semi-solid transport media. Sterile 7H9 (Difco Middlebrook, Becton Dickinson and Company) transport medium was prepared to contain 0.5% Agar-Agar (Merck), 0.2% glycerol (Sigma), 2% PANTA (Becton Dickinson and Company) and 10% OADC enrichment (Becton Dickinson and Company) [11]. Briefly after autoclaving the dissolved 7H9 powder and the agar, the glycerol, PANTA and OADC were added and 5 ml portions of the still warm medium was filled into empty cotton swab tubes (Copan). The transport medium was stored at 4°C until use. Following sample collection, swabs were inserted into the tube containing the transport medium and locally stored at 4°C whenever possible. Due to the remoteness of the BU endemic areas in which clinical samples for this study were collected, timely transport to adequately equipped laboratories with sufficient capacity was difficult. Therefore at 4–6 month intervals, samples were transported to the laboratory at the Swiss Tropical and Public Health Institute at ambient temperature. Once in the laboratory, swabs were stored at 4°C until processing.

### PCR, decontamination and primary inoculation

For DNA extraction and culturing, swabs were transferred to 14 mL McCartney glass bottles (Marienfeld, Germany) that were filled approximately to one third with 3 mm diameter glass beads (Marienfeld, Germany) and 2–5 mL of sterile phosphate buffered saline (PBS). The bottles were vortexed for 1.5 minutes and DNA was extracted from 1 mL of the solution as described by Lavender and Fyfe [15]. Extracts were analyzed in duplicate by IS2404 real-time PCR (qPCR) as previously described [15].

Decontamination of 1 mL of the remaining extract in PBS was performed with 1 mL of 1 M NaOH for 10 minutes (NaOH_10min), 1 mL of 1 M NaOH for 20 minutes (NaOH_20min), 1 mL of 5% OA for 30 minutes (OA_30min) or 1 mL of 5% OA for 1 hour (OA_1h) at room temperature with occasional vortexing. Some but not all extracts were decontaminated with multiple decontamination methods in parallel (Additional file 1: Table S1). Decontaminated extracts were diluted with 20 mL of sterile PBS. The decontaminated samples were then centrifuged for 30 minutes at 3000 g, the supernatant decanted and the pellet re-suspended in 0.15 to 0.25 mL of sterile PBS. Of the re-suspended pellet, 0.1 mL was transferred to Löwenstein-Jensen (LJ) medium slants with glycerol (Becton Dickinson and Company) or the same LJ medium slants supplemented with 2% PANTA (LJ_PANTA). As with the decontamination methods above, some but not all re-suspended pellets were inoculated on both media in parallel.

### Culture processing and *M. ulcerans*identification

All inoculated cultures were incubated at 30°C until *M. ulcerans* growth could be observed. Slants were regularly examined and discarded if contamination, i.e. overgrowth with other faster growing microorganisms, was detected. All inoculations with no growth were kept for a minimum of 25 weeks before discarding. Suspected *M. ulcerans* growth was confirmed by colony PCR using primers MU154 (5’-ggcagttacttcactgcaca-3’) and MU155 (5’-cggtgatcaagcgttcacga-3’) and amplification for 32 cycles of 30 seconds at 94°C, 30 seconds at 60°C and 1 minute at 72°C. PCR products were resolved in a 1.5% agarose gel.

### Statistical analysis

To identify factors that affect the rate of contamination and recovery of *M. ulcerans* primary cultures, three sets of univariate and multivariate statistical analyses, one with all inoculations and two with subsets, were performed. The factors investigated included: transport medium, decontamination method, inoculation media, swab qPCR Ct value, patient qPCR result, the time from sampling to inoculation and the number of weeks before diagnosis as reported by the patient.

The first analysis identified differences between inoculations that did not contaminate *versus* those that did result in contamination (non-contamination *vs*. contamination). This analysis aimed at identifying factors that affect contamination of cultures of samples taken from lesions clinically suspected of BU, independent of the presence of *M. ulcerans* on the swab. Specifically, the data set used for this analysis included both qPCR positive and negative swabs and inoculations with any of the three possible outcomes: *M. ulcerans* growth, contamination with another microorganisms or no growth. Swabs with a negative qPCR result in this data set were assigned a Ct value of 76.80 (reciprocal of the midpoint between zero and the minimum of 1/Ct). These qPCR negative swabs were included in the analysis to investigate general factors that affect contamination of wound exudates and if the presence of *M. ulcerans* affects the rate of contamination.

In the second analysis, a subset of inoculations was analyzed to identify factors that affect *M. ulcerans* growth (*M. ulcerans* growth *vs*. no growth). All inoculations with no realistic probability of resulting in *M. ulcerans* growth, i.e. contaminated inoculations and inoculations from swabs that were qPCR negative were excluded from this analysis.

Finally in the third analysis, we studied factors that affected the recovery of *M. ulcerans* if some inoculations resulted in contamination (*M. ulcerans* growth *vs*. contamination or no growth). This subset included all inoculations originating from qPCR positive swabs independent of their outcome, i.e. also including swabs for which growth may have been undetectable because of overgrowth by other microorganisms. In this third analysis, the outcomes “no growth” and “contamination” were grouped together as the undesired outcome.

**Figure 1:**
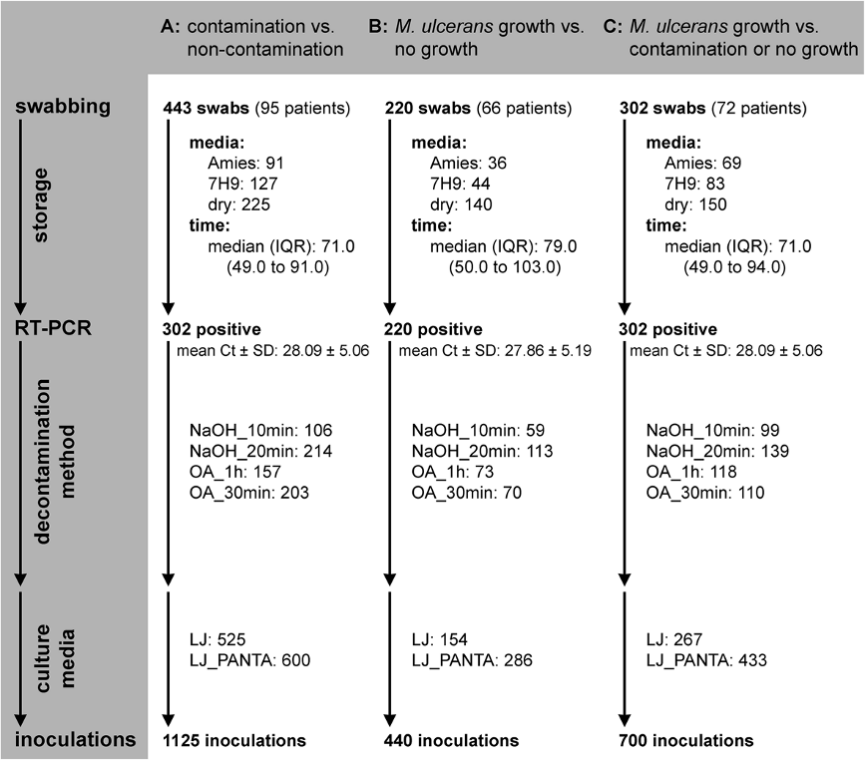
**Workflow for swabs included in the statistical analysis.** The complete set of *M. ulcerans* primary culturing inoculations was used to identify factors that affect the rate of contamination (non-contamination *vs*. contamination; **A**). A subset of the inoculations was used to identify factors that influence the growth of *M. ulcerans* in the absence of any contamination (*M. ulcerans* growth *vs*. no growth; **B**) and a second subset was used to identify factors that affect *M. ulcerans* growth if some of the inoculations resulted in contamination (*M. ulcerans* growth *vs*. contamination or no growth; **C**). The number of swabs collected as well as the transport media used and the storage time are shown. Further, the number of qPCR positive swabs with their average Ct value are given and the numbers of decontaminations as well as inoculations performed are shown. Finally the number of total inoculations in each of the data sets is indicated.

Workflows of the swabs included in the three data sets are shown in Figure 1 and the numbers of samples decontaminated with two decontamination methods in parallel are listed in Additional file 1: Table S1.

For the identification of factors that contribute to the outcomes studied, generalized linear mixed models with patient identification and identification of each individual swab as random effects were used. Factors that individually had a p-value of the association of less than 0.2 were included in the multivariate analyses. The software SAS (SAS Institute, Cary, USA; release 9.3), RStudio (RStudio, Boston, USA, version 0.95.262) and R (The R Foundation for Statistical Computing; version 2.15.1) were used to perform the analyses.

## Results

### Factors affecting the rate of contamination

In total 443 swabs, collected from 73 qPCR confirmed and 22 non-confirmed patients were included in the analysis for factors affecting the rate of contamination. Of all these swabs, 302 were qPCR positive with an average Ct value of 28.09 (Figure 1A). Of the 1125 culture inoculations from these swabs, 7.8% (88/1125) resulted in *M. ulcerans* growth. The 88 *M. ulcerans* culture positive inoculations originated from samples collected from 31 patients, which corresponds to a per patient positivity of 32.6% (31/95; Table 1). Further 52.5% (591/1125) of the inoculations yielded no growth and 39.6% (446/1125) resulted in contamination. The median time to detection of contamination was 5.0 (interquartile range (IQR) = 4.0 to 11.0) days (Table 1). One-to-one analysis was used to identify factors that should be included in the multivariate analysis (Additional file 1: Table S2). When studying the combined effect of factors on the rate of contamination, we found that the transport medium had an overall significant (p-value: 0.008) effect on the probability of contamination. Specifically, swabs transported dry had a 57.7 times lower probability of contamination compared to swabs transported in Amies medium (Table 2). We found no significant difference in terms of the rate of contamination between swabs transported in 7H9 or Amies medium. As also shown in Table 2, there was no overall significant difference (p-value: 0.266) in the rate of contamination between the four decontamination methods evaluated here. The inoculated culture medium on the other hand significantly (p-value: <0.001) influenced the rate of contamination, with cultures on LJ having a 3.8 (1/0.264) times higher probability of contamination than cultures inoculated onto LJ_PANTA. The multivariate analysis further showed that there was a significant (p-value: 0.007) interaction between transport medium and the number of days from sampling to inoculation (Table 2). Finally, both an increase in the Ct value of the swab (p-value: 0.236) and the storage time from sampling to inoculation (p-value: 0.606) did not have a significant effect on the rate of contamination of the cultures (Table 2). Overall, this analysis to identify conditions that were best suited to prevent contamination of *M. ulcerans* primary cultures suggested that swabs should be stored dry, any of the evaluated decontamination methods can be used, cultures should be inoculated onto LJ supplemented with PANTA and neither the Ct value of the IS2404 qPCR nor the time from sampling to inoculation had a significant effect on the rate of contamination.

**Table 1:** **Outcomes of** ***M. ulcerans*** **primary culturing**

Attribute		Non-contamination vs. contamination ^#^	*M. ulcerans* growth vs. no growth ^##^	*M. ulcerans* growth vs. contamination or no growth ^###^
*M. ulcerans* growth per swab (%)		71/443 (16.03)	71/220 (32.27)	71/302 (23.50)
*M. ulcerans* growth per decontamination procedure (%)	Total	80/680 (11.76)	80/315 (25.40)	80/466 (17.17)
	NaOH_10min	21/106 (19.81)	21/59 (35.59)	21/99 (21.21)
	NaOH_20min	19/214 (8.88)	19/113 (16.81)	19/139 (13.67)
	OA_1h	17/157 (10.83)	17/73 (23.29)	17/118 (14.41)
	OA_30min	23/203 (11.33)	23/70 (32.86)	23/110 (20.91)
*M. ulcerans* growth per inoculation (%)		88/1125 (7.82)	88/440 (20.00)	88/700 (12.57)
*M. ulcerans* growth per patient (%)		31/95 (32.63)	31/66 (46.97)	31/72 (43.06)
Contamination per swab (%)		284/443 (64.11)	-	188/302 (62.25)
Contamination per decontamination procedure (%)	Total	346/680 (50.88)	-	223/466 (47.85)
	NaOH_10min	54/106 (50.94)	-	48/99 (48.48)
	NaOH_20min	97/214 (45.33)	-	58/139 (41.73)
	OA_1h	74/157 (47.13)	-	54/118 (45.76)
	OA_30min	121/203 (59.61)	-	63/110 (57.27)
Contamination per inoculation (%)		446/1125 (39.64)	-	260/700 (37.14)
No growth (%)		591/1125 (52.53)	352/440 (80.00)	352/700 (50.29)
Days to primary outcome*		5.0 (4.0; 11.0)	67.0 (55.0; 105.2)	67.0 (55.0; 105.2)

^#^Analysis of 1125 inoculations from 95 patients with contamination as the primary outcome.

^##^Analysis of 440 inoculations from 66 patients with *M. ulcerans* growth as the primary outcome.

^###^Analysis of 700 inoculations from 72 patients with *M. ulcerans* growth as the primary outcome.

*Median with IQR in parentheses.

**Table 2:** **Association** ^**§**^ **between non-contamination and all factors of interest**

Factor	Value of factor	Odds ratio ^§^	95% CI odds ratio	Overall effect p-value
**Transport medium** ^**§§**^	7H9	1.579	0.632 - 3.944	0.008
	dry	57.675	23.704 - 140.334	
	Amies (ref. level)	1.000		
**Decontamination medium**	OA_1h	0.715	0.362 - 1.412	0.266
	OA_30min	0.540	0.260 - 1.122	
	NaOH_20min	0.879	0.383 - 2.016	
	NaOH_10min (ref. level)	1.000		
**Inoculation media** ^**§§**^	LJ	0.264	0.162 - 0.429	<0.001
	LJ_PANTA (ref. level)	1.000		
**Swab qPCR Ct value**	for an increase in 1 unit Ct	0.992	0.978 - 1.006	0.236
**Time from sampling to inoculation**	for an increase in 10 days			0.606
**Interaction of transport medium and days from sampling to inoculation**	when 7H9^§§§^	0.982	0.824 - 1.171	0.007
	when dry^§§§^	1.167	1.032 - 1.321	
	when Amies^§§§^ (ref. level)	0.799	0.643 - 0.993	

^§^Adjusted for random effects of the patient and swab.

^§§^An interaction (p-value: <0.001) between transport medium and inoculation media was observed.

^§§§^For an increase in 10 days from the mean number of days from sampling to inoculation.

### Factors affecting the rate of *M. ulcerans*recovery in primary culturing

To identify factors that affect the recovery of *M. ulcerans*, 440 inoculations from 220 qPCR positive swabs originating from 66 qPCR positive patients were analyzed. Only the subset of swabs with a realistic probability of resulting in *M. ulcerans* growth, as defined in Material and Methods above, were used for this analysis. The average qPCR Ct value of the swabs was 27.9. Of all the inoculations included in the analysis, 88 inoculations of exudates collected from 31 patients resulted in *M. ulcerans* growth and the rest yielded no growth. This corresponds to a per inoculation positivity rate of 20.0% (88/440) and a per patient positivity rate of 47.0% (31/66). Because it was not possible to determine if the contaminated inoculations could have resulted in *M. ulcerans* growth, they were not included in this analysis (Materials and Methods; Table 1). The median time to detectable *M. ulcerans* growth was 67.0 (IQR = 55.0 to 105.2) days (Table 1). One-to-one analyses were again used to identify factors to be included in the multivariate analysis (Additional file 1: Table S3). In the multivariate analysis, we found that the transport medium had an overall significant effect (p-value: <0.001) on the probability of *M. ulcerans* growth (Table 3). Specifically, swabs transported dry had a 97.7% reduced chance (p-value <0.001) of *M. ulcerans* growth compared to samples transported in Amies medium (Table 3). Between swabs transported in Amies or 7H9 medium no significant difference was found, although transport in Amies medium had a tendency to increase the chance of *M. ulcerans* recovery (odds ratio for transport in 7H9: 0.304; Table 3). Further in the multivariate analysis there was no significant difference in the chance of *M. ulcerans* recovery between the decontamination methods evaluated (p-value: 0.519) and inoculation on either LJ or LJ_PANTA (p-value: 0.216). However, with an increase of the qPCR Ct value by one unit, the probability of *M. ulcerans* growth was reduced by 12.1% (p-value: 0.044; Table 3, 1 - OR) and with every 10 extra days of storage between sampling and inoculation, the probability of *M. ulcerans* growth decreased significantly (p-value: 0.001) by 45.9%. Our analysis further detected a moderately significant (p-value: 0.074) interaction between the transport time and the transport medium, with Amies medium having the best chance of *M. ulcerans* growth for an increase in transport time by 10 days.

**Table 3:** **Association** ^**§**^ **between** ***M. ulcerans*** **growth and all factors of interest with a relevant effect**

Factor	Value of factor	Odds ratio ^§^	95% CI odds ratio	Overall effect p-value
**Transport medium**	7H9	0.304	0.046 - 2.026	<0.001
	dry	0.023	0.004 - 0.143	
	Amies (ref. level)	1.000		
**Decontamination medium**	OA_1h	0.414	0.102 - 1.683	0.519
	OA_30min	0.697	0.127 - 3.837	
	NaOH_20min	1.242	0.204 - 7.546	
	NaOH_10min (ref. level)	1.000		
**Inoculation media**	LJ	0.478	0.148 - 1.545	0.216
	LJ_PANTA (ref. level)	1.000		
**Swab qPCR Ct value**	for an increase in 1 unit Ct	0.879	0.775 - 0.996	0.044
**Time from sampling to inoculation**	for an increase in 10 days			0.001
**Interaction of transport medium and days from sampling to inoculation**	when 7H9^§§^	0.531	0.320 - 0.881	0.074
	when dry^§§^	0.880	0.704 - 1.099	
	when Amies^§§^ (ref. level)	0.541	0.347 - 0.844	

^§^Adjusted for random effects of the patient and swab.

^§§^For an increase in 10 days from mean number of days from sampling to inoculation.

Overall, the analysis to evaluate factors that affect the recovery of *M. ulcerans* revealed that: i) storage in either Amies or 7H9 medium was significantly better than keeping swabs dry; ii) none of the decontamination methods tested had a significantly better effect on the growth of *M. ulcerans* and iii) the *M. ulcerans* recovery was not affected by inoculation of samples onto media containing PANTA. On the other hand, the analysis showed that both a one unit increase in the Ct value of the IS2404 qPCR and a 10 day increase in the turnover time of the samples had a significantly negative effect on the rate of *M. ulcerans* recovery.

### *M. ulcerans*recovery versus no growth or contamination

**Figure 2:**
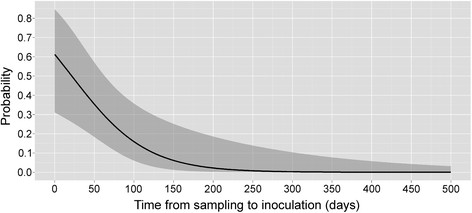
**Predicted probabilities for** ***M. ulcerans*** **growth.** Based on the *M. ulcerans* growth *vs*. no growth or contamination model the probability of *M. ulcerans* growth was predicted as a function of transport time for samples transported in Amies medium, decontaminated with NaOH for 10 minutes, inoculated onto LJ medium supplemented with PANTA and if the Ct value of the qPCR was 27.8. Mean predicted probability of the *M. ulcerans* growth rate and 95% confidence intervals are shown.

For the identification of factors that affect *M. ulcerans* recovery in a scenario where some cultures are contaminated, 700 inoculated cultures from 302 qPCR positive swabs (average qPCR Ct value of 28.09) taken from 72 qPCR positive patients were analyzed. As shown in Table 1, 88 inoculations collected from 31 patients of the total 700 inoculations resulted in *M. ulcerans* growth. This corresponded to a per patient positivity rate of 43.1%. Further, 37.1% (260/700) of the inoculations were contaminated and 50.3% (352/700) did not result in any growth. Analysis of these inoculations in a multivariate analysis showed, that transport medium had a significant effect (p-value: 0.019) on the recovery of *M. ulcerans* (Table 4). Specifically, swabs transported in Amies medium showed the best recovery rate of *M. ulcerans*, although not significantly better than 7H9 medium (95% CI of OR: 0.269 - 1.415). As also seen in the analysis for non-contamination (Table 2) and *M. ulcerans* growth (Table 3), the decontamination methods evaluated here did not significantly differ in their effect on the chance of *M. ulcerans* recovery (p-value: 0.295; Table 4). On the other hand the use of the inoculated media had a significant (p-value: 0.003) impact on the chance of *M. ulcerans* recovery, with the use of LJ as compared to LJ_PANTA reducing the probability of *M. ulcerans* recovery by 65.5%. As further shown in Table 4, a one unit increase in the qPCR Ct value away from the mean qPCR Ct value of the inoculated swabs decreased the chance of *M. ulcerans* recovery by 10.8% (p-value: 0.011). Furthermore, consistent with the analysis of factors affecting *M. ulcerans* growth (Table 3), the time span from sampling to inoculation significantly (p-value: 0.006) reduced the chance of *M. ulcerans* isolation, with a decrease of 19.1% for every 10 extra days of storage compared to the mean storage time of 80.2 days (Table 4, Figure 2). As shown in Figure 2, the predicted probability of *M. ulcerans* recovery decreased from 61.2% at zero days of storage to 26.8% within 70 days of storage. Specifically for swabs with a qPCR Ct value of 27.8, that were transported in Amies medium, decontaminated using NaOH for 10 minutes and inoculated onto LJ with PANTA, a probability of *M. ulcerans* growth of 61.2% can be expected if samples were stored for zero days. If the samples are stored for 50 or 100 days, the predicted probability decreases to 35.3% and 15.9% respectively.

**Table 4:** **Model describing the association** ^**§**^ **between** ***M. ulcerans*** **growth versus no growth or contamination and all factors of interest with a relevant effect**

Factor	Value of factor	Odds ratio ^§^	95% CI odds ratio	Overall effect p-value
**Transport medium**	7H9	0.617	0.269 - 1.415	0.019
	dry	0.248	0.094 - 0.655	
	Amies (ref. level)	1.000		
**Decontamination medium**	OA_1h	0.440	0.182 - 1.062	0.295
	OA_30min	0.529	0.203 - 1.375	
	NaOH_20min	0.739	0.236 - 2.313	
	NaOH_10min (ref. level)	1.000		
**Inoculation media**	LJ	0.345	0.169 - 0.703	0.003
	LJ_PANTA (ref. level)	1.000		
**Swab qPCR Ct value**	for an increase in 1 unit Ct	0.892	0.818 - 0.974	0.011
**Time from sampling to inoculation**	for an increase in 10 days	0.809	0.695 - 0.941	0.006

^§^Adjusted for random effects of the patient and swab.

Overall our analysis of the sample set consisting of only qPCR positive swabs but including all possible outcomes of the inoculations, showed that either of the moist transport media increased the chance of *M. ulcerans* recovery compared to samples kept dry, none of the decontamination methods yielded superior results and *M. ulcerans* culturing was favored on PANTA supplemented LJ medium. Further the analysis showed that both a one unit increase in the Ct value of the IS2404 qPCR and a 10 day increase in the turnover time of the samples negatively impacted the chance of *M. ulcerans* isolation.

## Discussion

In the present study we evaluated the effect of various factors on the rate of *M. ulcerans* primary isolation from clinical specimens to develop a method for *M. ulcerans* recovery from samples collected from very remote BU endemic areas. For this, we examined three sets of inoculations, i) to identify factors that reduce the rate of contamination of the primary cultures, ii) to determine which factors increase the rate of *M. ulcerans* recovery in a scenario where none of the cultures are contaminated and iii) to evaluate the effect of factors in the realistic setting where some *M. ulcerans* growth will be missed due to the contamination of cultures.

In a study on IS2404 PCR positive tissue biopsies which were stored for up to 26 weeks in semi-solid transport medium, Eddyani *et al*. were able to achieve a culture positivity rate of 45.2%. This showed that the establishment of an *M. ulcerans* strain collection from remote BU endemic areas from tissue samples is possible [11]. Other studies using either tissue biopsies or swab and FNA samples and shorter transport times have also reported culture positivity rates of about 50% with the rest of the cases, although clinically diagnosed and PCR confirmed, remaining culture negative [6],[13],[16],[17]. In a study comparable to the present analysis, Eddyani *et al*. cultured *M. ulcerans* from FNA that were stored for two weeks before processing and achieved a per sample positivity rate of 17.6% [12]. In spite of long storage intervals prior to processing, we have achieved here a per PCR–reconfirmed-sample positivity rate of 23.5%, corresponding to a PCR-reconfirmed-patient *M. ulcerans* culture positivity rate from swab and FNA samples of 43.1%. Given the similarity in these *M. ulcerans* recovery rates in several studies employing different clinical specimens and approaches, the reason for lack of growth of *M. ulcerans* from certain patients should be further investigated [18].

To increase *M. ulcerans* recovery rates, Yeboah-Manu *et al.* have suggested collecting and processing multiple swab or FNA samples per patient. To increase the chance of culture positivity, we have ensured that lesion exudates were collected from all around ulcers. Further, we collected and processed several (median = 4; IQR = 2 to 6) swabs from most patients and this repeated sampling did indeed affect the rate of recovery of *M. ulcerans* per patient*.* Only considering qPCR positive swabs, we observed a significant difference (p-value 0.003) in the number of swabs collected from culture positive patients (median: 5; IQR: 3 to 6 swabs) versus the number of swabs collected from culture negative patients (median: 2; IQR: 1 to 5 swabs). Based on these results, we recommend the collection of up to five swab samples per patient prior to treatment start to increase the probability of recovering the infecting *M. ulcerans* strain.

In both our analyses for *M. ulcerans* growth and for contamination of cultures, there was no significant difference between swabs transported in 7H9 or Amies medium. However, based on the lower costs of Amies transport medium compared to the 7H9 medium (approximately 0.7 USD per Amies swab and 2.8 USD per 7H9 swab with PANTA and OADC), we recommend the use of Amies medium for the transport of swabs collected from BU patients.

In our analysis, the number of days for which samples were stored did not significantly affect the rate of contamination of the inoculated cultures but longer storage did significantly reduce the rate of *M. ulcerans* recovery (Tables 3 and 4, Figure 2). This is contrary to a previous report of culturing from tissue biopsies which found that that storage time did not affect the rate of *M. ulcerans* recovery [11]. *M. ulcerans* may thus survive better if transported in the context of a tissue biopsy than as swab sample. As can be expected, the Ct value of the IS2404 qPCR had a significant effect on the rate of *M. ulcerans* recovery (Tables 3 and 4). On the other hand, neither the qualitative nor the quantitative qPCR result of a swab nor the BU status, i.e. the overall patient qPCR result , had a significant effect on the rate of contamination of the primary cultures in the one-by-one or the multivariate analysis (Table 2 and Additional file 1: Table S2). In line with a recent report on secondary infections of BU lesions [19], this finding does not support the assumption that BU lesions are less prone to contamination with secondary microorganisms than other wounds [20].

In our analysis there was no significant difference between the four decontamination methods evaluated, although in the univariate analysis for *M. ulcerans* growth, NaOH for 10 minutes appeared to outperform the other decontamination options with borderline significance (p-value: 0.076, Additional file 1: Table S3). Since 10 min NaOH is also the quickest decontamination method, we therefore suggest this one to be used.

Our analysis of *M. ulcerans* growth vs. contamination or no growth (Figure 1C), showed that the use of LJ_PANTA compared to LJ had a significant positive effect (p-value: 0.003) on the rate of recovery of *M. ulcerans*. This is similar to what has been found by Yeboah-Manu *et al*. where *M. ulcerans* recovery was also significantly improved (p-value <0.001) on LJ_PANTA compared to LJ alone [13].

By evaluating a small set of swabs, Yeboah-Manu *et al.* have previously been able to show that culturing from dry cotton swabs is feasible [13]. In our study we have confirmed this finding. However the chance of *M. ulcerans* recovery from swabs stored dry is reduced by 75.2% compared to swabs transported in Amies (Table 4) and in our analysis dry swabs only achieved a per patient culture positivity rate of 13.1%.

## Conclusions

In conclusion, our results show that primary culturing of *M. ulcerans* from cotton swabs after long turnover time is possible. Based on our findings we suggest that this type of samples should be transported in Amies medium, that they should be decontaminated in 0.5 M NaOH for 10 minutes and that cultures should be inoculated onto LJ medium supplemented with 2% PANTA. Furthermore, multiple samples (approximately 5) should be collected from each patient and only the PCR positive swabs should be inoculated for culturing.

Overall, the here identified method can help to establish *M. ulcerans* strain collections from very remote BU endemic areas. An increased number of available *M. ulcerans* strains from all endemic areas will be a valuable resource for studies to increase our understanding of pathology, transmission and many other aspects of BU.

## Additional file

## Electronic supplementary material

Additional file 1: Table S1.: Comparison of decontamination methods showing the number of samples decontaminated in parallel with the decontamination methods indicated. **Table S2.** Results of univariate association between non-contamination of *M. ulcerans* cultures and each of the factors of interest. **Table S3.** Results of univariate association between *M. ulcerans* growth and each of the factors of interest. (PDF 33 KB)

Below are the links to the authors’ original submitted files for images.

Authors’ original file for figure 1

Authors’ original file for figure 2

## References

[1] Merritt RW, Walker ED, Small PLC, Wallace JR, Johnson PDR, Benbow ME, Boakye DA (2010). Ecology and transmission of Buruli ulcer disease: a systematic review. PLoS Negl Trop Dis.

[2] Walsh DS, Portaels F, Meyers WM (2011). Buruli ulcer: advances in understanding Mycobacterium ulcerans infection. Dermatol Clin.

[3] WHO | Provisional guidance on the role of specific antibiotics in the management of *Mycobacterium ulcerans* disease (Buruli ulcer).[], [http://www.who.int/buruli/information/antibiotics/en/index.html]

[4] Buruli ulcer: diagnosis of Mycobacterium ulcerans disease: a manual for health care providers. In Edited by Portaels F, Johnson P, Meyers WM.[], [http://apps.who.int/iris/handle/10665/67000]

[5] Yeboah-Manu D, Bodmer T, Mensah-Quainoo E, Owusu S, Ofori-Adjei D, Pluschke G (2004). Evaluation of decontamination methods and growth media for primary isolation of Mycobacterium ulcerans from surgical specimens. J Clin Microbiol.

[6] Portaels F, Agular J, Fissette K, Fonteyne PA, De Beenhouwer H, de Rijk P, Guédénon A, Lemans R, Steunou C, Zinsou C, Dumonceau JM, Meyers WM (1997). Direct detection and identification of Mycobacterium ulcerans in clinical specimens by PCR and oligonucleotide-specific capture plate hybridization. J Clin Microbiol.

[7] Röltgen K, Qi W, Ruf M-T, Mensah-Quainoo E, Pidot SJ, Seemann T, Stinear TP, Käser M, Yeboah-Manu D, Pluschke G (2010). Single nucleotide polymorphism typing of Mycobacterium ulcerans reveals focal transmission of buruli ulcer in a highly endemic region of Ghana. PLoS Negl Trop Dis.

[8] Beissner M, Awua-Boateng N-Y, Thompson W, Nienhuis WA, Klutse E, Agbenorku P, Nitschke J, Herbinger K-H, Siegmund V, Fleischmann E, Adjei O, Fleischer B, van der Werf TS, Loscher T, Bretzel G (2010). A genotypic approach for detection, identification, and characterization of drug resistance in Mycobacterium ulcerans in clinical samples and isolates from Ghana. Am J Trop Med Hyg.

[9] Petroff SA (1915). A new and rapid method for the isolation and cultivation of tubercle bacilli directly from the sputum and feces. J Exp Med.

[10] Palomino JC, Portaels F (1998). Effects of decontamination methods and culture conditions on viability of Mycobacterium ulcerans in the BACTEC system. J Clin Microbiol.

[11] Eddyani M, Debacker M, Martin A, Aguiar J, Johnson CR, Uwizeye C, Fissette K, Portaels F (2008). Primary culture of Mycobacterium ulcerans from human tissue specimens after storage in semisolid transport medium. J Clin Microbiol.

[12] Eddyani M, Fraga AG, Schmitt F, Uwizeye C, Fissette K, Johnson C, Aguiar J, Sopoh G, Barogui Y, Meyers WM, Pedrosa J, Portaels F (2009). Fine-needle aspiration, an efficient sampling technique for bacteriological diagnosis of nonulcerative Buruli ulcer. J Clin Microbiol.

[13] Yeboah-Manu D, Danso E, Ampah K, Asante-Poku A, Nakobu Z, Pluschke G (2011). Isolation of Mycobacterium ulcerans from swab and fine-needle-aspiration specimens. J Clin Microbiol.

[14] Bratschi MW, Bolz M, Minyem JC, Grize L, Wantong FG, Kerber S, Njih Tabah E, Ruf M-T, Mou F, Noumen D, Um Boock A, Pluschke G (2013). Geographic distribution, age pattern and sites of lesions in a cohort of buruli ulcer patients from the mapé basin of cameroon. PLoS Negl Trop Dis.

[15] Lavender CJ, Fyfe JAM (2013). Direct detection of Mycobacterium ulcerans in clinical specimens and environmental samples. Methods Mol Biol Clifton NJ.

[16] Beissner M, Herbinger K-H, Bretzel G (2010). Laboratory diagnosis of Buruli ulcer disease. Future Microbiol.

[17] Mensah-Quainoo E, Yeboah-Manu D, Asebi C, Patafuor F, Ofori-Adjei D, Junghanss T, Pluschke G (2008). Diagnosis of Mycobacterium ulcerans infection (Buruli ulcer) at a treatment centre in Ghana: a retrospective analysis of laboratory results of clinically diagnosed cases. Trop Med Int Health TM IH.

[18] Williamson H, Phillips R, Sarfo S, Wansbrough-Jones M, Small P (2014). Genetic diversity of PCR-positive, culture-negative and culture-positive mycobacterium ulcerans isolated from buruli ulcer patients in Ghana. PLoS One.

[19] Yeboah-Manu D, Kpeli GS, Ruf M-T, Asan-Ampah K, Quenin-Fosu K, Owusu-Mireku E, Paintsil A, Lamptey I, Anku B, Kwakye-Maclean C, Newman M, Pluschke G (2013). Secondary bacterial infections of buruli ulcer lesions before and after chemotherapy with streptomycin and rifampicin. PLoS Negl Trop Dis.

[20] Demangel C, Stinear TP, Cole ST (2009). Buruli ulcer: reductive evolution enhances pathogenicity of Mycobacterium ulcerans. Nat Rev Microbiol.

