# Supplementary material for: Primary cultivation: factors affecting contamination and Mycobacterium ulcerans growth after long turnover time of clinical specimens
Source: BMC Infect Dis. 2014 Nov 30;14:636. doi: 10.1186/s12879-014-0636-7 (PMC4264541; doi:10.1186/s12879-014-0636-7)
Supplement: Supplementary file 1 — Additional file 1: Table S1.: Comparison of decontamination methods showing the number of samples decontaminated in parallel with the decontamination methods indicated. Table S2. Results of univariate association between non-contamination of M. ulcerans cultures and each of the factors of interest. Table S3. Results of univariate association between M. ulcerans growth and each of the factors of interest. (PDF 33 KB) [file 12879_2014_636_MOESM1_ESM.pdf]

Additional Table 1: Number of samples decontaminated with multiple decontamination methods in parallel

Decontamination method*	Non-contamination vs. contamination	*M. ulcerans* growth vs. no growth	*M. ulcerans* growth vs. contamination or no growth
**NaOH_10 and OA_30min**	19	3	13
**NaOH_10 and OA_1h**	87	44	86
**NaOH_20 and OA_30min**	61	25	33
**OA_1h and OA_30min**	70	23	32

* Number of clinical samples decontaminated with the combinations of decontamination methods indicated for the three set of samples analysed.

Additional Table 2: Association[§] between non-contamination of *M. ulcerans* cultures and each of the factors of interest

Factor	Value of factor	Odds ratio[§]	95% CI odds ratio	Overall effect (p-value)
**Transport Medium**	7H9	0.840	0.462 - 1.527	<0.001
	dry	24.314	13.268 - 44.555	
	Amies (ref. level)	1.000		
**Decontamination medium**	OA_1h	0.853	0.465 - 1.567	0.021
	OA_30min	0.749	0.396 - 1.414	
	NaOH_20min	1.777	0.860 - 3.673	
	NaOH_10min (ref. level)	1.000		
**Inoculation media**	LJ	0.441	0.307 - 0.634	<0.001
	LJ_PANTA (ref. level)	1.000		
**Swab qPCR Ct value**	for an increase in 1 unit Ct	0.990	0.978 - 1.002	0.119
**Patient qPCR result**	negative	0.799	0.379 - 1.682	0.554
	positive (ref. level)	1.000		
**Time from sampling to inoculation**	for an increase in 10 day	1.402	1.282 - 1.534	<0.001
**Weeks before diagnosis***	for an increase by 1 week	0.992	0.976 - 1.007	0.285

[§] Adjusted for random effects of the patient and swab identification.

* Information as reported by the patient.

Additional Table 3: Association[§] between *M. ulcerans* growth and each of the factors of interest.

Factor	Value of factor	Odds ratio[§]	95% CI odds ratio	Overall effect p-value
**Transport medium**	7H9	0.343	0.099 - 1.192	<0.001
	dry	0.004	0.001 - 0.015	
	Amies (ref. level)	1.000		
**Decontamination medium**	OA_1h	0.373	0.135 - 1.032	0.076
	OA_30min	0.396	0.136 - 1.157	
	NaOH_20min	0.229	0.071 - 0.734	
	NaOH_10min (ref. level)	1.000		
**Inoculation media**	LJ	0.510	0.239 - 1.089	0.082
	LJ_PANTA (ref. level)	1.000		
**Swab qPCR Ct value**	for an increase in 1 unit Ct	0.947	0.868 - 1.033	0.216
**Time from sampling to inoculation**	for an increase in 10 day	0.621	0.522 - 0.738	<0.001
**Number of days of treatment before sampling**	for an increase in 1 day	1.033	0.965 - 1.106	0.343
**Weeks before diagnosis***	1 week	1.002	0.976 - 1.029	0.865

[§] Adjusted for random effects of the patient and swab identification.

* Information as reported by the patient.
