# Supplementary figures and images for: Primary cultivation: factors affecting contamination and Mycobacterium ulcerans growth after long turnover time of clinical specimens
Source: BMC Infect Dis. 2014 Nov 30;14:636. doi: 10.1186/s12879-014-0636-7 (PMC4264541; doi:10.1186/s12879-014-0636-7)

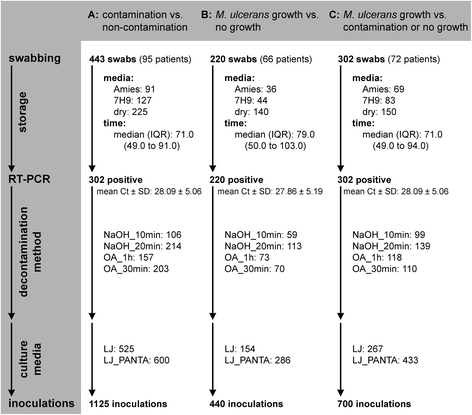

Supplement: Supplementary file 2 — Authors’ original file for figure 1 [file 12879_2014_636_MOESM2_ESM.gif]

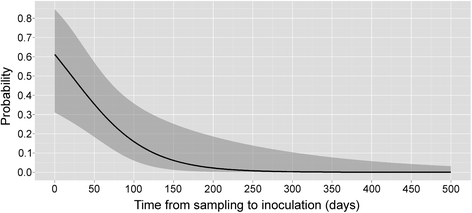

Supplement: Supplementary file 3 — Authors’ original file for figure 2 [file 12879_2014_636_MOESM3_ESM.gif]
